# Clinical aspects of Emery-Dreifuss muscular dystrophy

**DOI:** 10.1080/19491034.2018.1462635

**Published:** 2018-04-10

**Authors:** Agnieszka Madej-Pilarczyk

**Affiliations:** Neuromuscular Unit, Mossakowski Medical Research Centre, Polish Academy of Sciences, Warsaw, Poland

**Keywords:** Emery-Dreifuss muscular dystrophy, lamin A/C, emerin, laminopathy, cardiomyopathy

## Abstract

Emery-Dreifuss muscular dystrophy (EDMD), clinically characterized by scapulo-humero-peroneal muscle atrophy and weakness, multi-joint contractures with spine rigidity and cardiomyopathy with conduction defects, is associated with structural/functional defect of genes that encode the proteins of nuclear envelope, including lamin A and several lamin-interacting proteins. This paper presents clinical aspects of EDMD in context to causative genes, genotype-phenotype correlation and its emplacement within phenotypic spectrum of skeletal muscle diseases associated with envelopathies.

## Introduction

Emery-Dreifuss muscular dystrophy (EDMD), clinically characterized scapulo-humero-peroneal muscle atrophy and weakness, multi-joint contractures with spine rigidity and cardiomyopathy with conduction defects, belongs to so-called envelopathies, a rare inherited human diseases with estimated incidence 3:1000000 (Orphanet Report Series, http://www.orpha.net). They are associated with structural/functional defect of genes that encode the proteins of nuclear envelope, i.e. lamins and lamin-interacting proteins [1]. Lamin A/C, encoded by *LMNA* gene, which is associated with subgroup of envelopathies called laminopathies [2], is a main component of nuclear lamina, located under the inner nuclear membrane, but in a smaller amount it is also found in the nucleoplasm [3]. Through interactions with chromatin and transcription factors, lamin A takes part in chromatin organization, DNA replication and transcription, and regulation of the cell cycle [4]. It binds to so-called LEM domain proteins, i.e. lamina-associated polypeptide (LAP), emerin and MAN1, thus via interaction with barrier to autointegration factor (BAF) [5], acts as structural integrator responsible for the maintenance of nuclear shape and stability, as well as for resistance to mechanical stress, which is extremely important for tissues exposed to it, e.g. skeletal and/or cardiac muscles. Another nuclear membrane proteins, which co-localize and bind lamin A and emerin, are transmembrane protein 43 (TMEM43, also called LUMA) encoded by *TMEM43* gene [6] and nesprins, i.e. nesprin-1 and nesprin-2, encoded by *SYNE1* and *SYNE2* genes [7], respectively. TMEM43 is also involved in organization of nuclear membrane [6], while nesprins, which are expressed at high levels in skeletal muscle, warrant proper positioning and anchorage of nuclei in multinucleated muscle cells during muscle development [8]. Lamin A/C is expressed in mature myocytes, in skeletal muscle stem cells and in satellite cells responsible for muscle regeneration, and through interactions with emerin and actin it modulates myoblast differentiation [9]. Mutations in the *LMNA* and in some of the genes listed above, i.e. *EMD*, *SYNE1*, *SYNE2* and *TMEM43*, as well as *FHL-1* gene, encoding a four and a half LIM domains protein 1 (FHL1), which does not belong to nuclear envelope proteins, but is expressed in striated muscles and probably takes part in sarcomere assembly, are associated with pathogenesis of skeletal muscle diseases, characterized by various clinical course and intra- and inter-familial variability, including Emery-Dreifuss muscular dystrophy (EDMD), which are to be discussed below [10–14]. Mutations in genes causative for EDMD, but also for other skeletal muscle phenotypes associated with nuclear envelope defects, lead to the damage and degeneration of myocytes. Concomitant expression of mutated lamin A/C in satellite cells impairs regeneration and differentiation of myocytes, leading to progression of muscle disease [15]. Nuclear defects in lamin-dependent muscle pathology include irregularity and loss of nuclear membrane, which may lead to nucleoplasm leakage, heterochromatin decondensation and its detachment from nuclear lamina, pseudoinclusions and, finally, nuclear fragmentation [16,17].

### Emery-Dreifuss muscular dystrophy associated with emerinopathy

Historically the first described envelopathy associated with skeletal muscle pathology was not a laminopathy, but an emerinopathy – Emery-Dreifuss muscular dystrophy, further called type 1 (EDMD1) (OMIM#310300), characterized by the X-linked recessive inheritance. Its clinical description was made at first by Cestan and Lejonne in 1902 [18]. Report of Dreifuss and Hogan from sixties of the XX century indicated slow progression of the disease and its non-disabling nature, which allowed to preserve everyday activities in majority of the patients [19]. In 1966 Emery and Dreifuss provided a detailed characteristics of EDMD1 [20]. The first symptoms of the disease, typically ankle and/or elbow contractures, are usually visible already in the first decade of life. They tend to be more evident and troublesome during adolescence growth spurt. Elbow contractures are usually more pronounced in dominant hand. Elongation of Achilles tendon is usually helpful, however sometimes the procedure should be repeated to achieve satisfactory result. Surgical correction of elbow contractures is more complicated and very often its effects are temporary. Muscle atrophy and weakness appear in the second decade of life, then slowly progresses, but usually does not affect walking ability. In EDMD1 skeletal muscle symptoms usually precede cardiac disease, which initially manifests as sinus bradycardia, supraventricular extrasystolic beats, atrioventricular blocks (AVB) of various grade, paroxysmal atrial fibrillation or flutter, tachycardia-bradycardia syndrome, finally leading to atrial standstill and possible gradual development of cardiomyopathy [19,20]. Conduction defect/arrhythmia may be either asymptomatic or result in mild/moderate and unspecific clinical manifestation, such as feeling of slow heart beating or palpitation, headache, vertigo, nightmares, fatigue, or be severe leading to syncope or even sudden death as a result of complete AVB. Majority of EDMD1 patients require pacemaker implantation as a primary prevention of SCD. There were reports of ventricular tachycardia in EDMD1, therefore regular monitoring in EDMD1 patients is highly recommended, as they might require ICD implantation [21]. The full clinical picture of EDMD1 with skeletal muscle symptoms and cardiac involvement is seen only in men. Twenty percent of female carriers may develop conduction defects, however muscle symptoms are very rare in them [22–24]. EDMD1 is associated with mutations in *EMD* gene, encoding emerin, with its *locus* mapped on q27-28 region of chromosome X [25,26]. In a majority of EDMD1 patients small out of frame deletions or splice-site mutations are found, while the remaining cases bear either a nonsense/missense mutation or large deletions [www.umd.be/EMD/]. Recurrent mutations are most often located in exons 1 and 2 of the *EMD* gene, in codons 1 or 34, respectively [27-29].

### Emery-Dreifuss muscular dystrophy associated with laminopathy

In the nineties of XX century it was concluded that EDMD can also result from abnormalities of the *LMNA* gene (EDMD2, OMIM#181350 with autosomal dominant and EDMD3, OMIM#616516 with autosomal recessive trait of inheritance) [30,31]. EDMD2 is most often associated with heterozygous missense mutations in the *LMNA* gene and expression of a mutated lamin A/C, which probably exerts dominant-negative toxic effect. The remaining cases of EDMD2 depend on deletions/duplications/nonsense mutations in *LMNA* gene, which result in loss of function of the encoded proteins [32–34]. The *LMNA* gene mutations in EDMD2 are disseminated randomly throughout exons 1–11 with no evident hot spot, however some recurrent mutations are known, such as these in codons 377 and 453. Majority of EDMD2 patients are sporadic cases, with *de novo* mutation in the *LMNA* gene [27,35]. Skeletal muscle symptoms in EDMD2 are less typical than in EDMD1. Some patients present milder phenotype with later onset and slow progression of muscle weakness and joint contractures [36,37], while others are affected with severe generalized muscle atrophy/weakness and joint contractures, and are at great risk of loss of independent walking. Paraspinal ligaments are frequently affected and together with posterior cervical muscle contractures can lead to permanent backward position of patient's head. Some patients have swallowing problems, probably as a result of cervical spine rigidity changing anatomical relations of the neck structures, however autonomic neuropathy cannot be excluded. Weakness of the respiratory muscles and chest deformity may cause respiratory failure. Cardiac involvement in EDMD2 is more variable and more difficult to predict than in EDMD1, as is the sequence of muscle and cardiac symptoms. In patients with mild muscle symptoms who develop arrhythmia or cardiac failure, cardiologist is sometimes the first specialist who combines heart and muscle symptomatology and refers them for neurological and genetic consultation [37]. Conduction defects and supraventricular arrhythmia is common for both forms of EDMD, however in EDMD2 dilated (sometimes restrictive) cardiomyopathy with impaired contractility of the left ventricle and decreased ejection fraction often develops, being a cause of fast-progressing cardiac failure, which may require heart transplantation [27,30,38]. In patients ineligible for heart transplantation due to respiratory failure or chest deformation, general condition may be further deteriorated by the right ventricle dysfunction and tricuspid valve insufficiency resulting from widening of the valve ring. Patients with EDMD2 are at high risk of life-threatening malignant ventricular arrhythmia [39], which may precede development of cardiomyopathy. The independent risk factors of malignant ventricular arrhythmias in a large cohort of *LMNA* mutation carriers included non-sustained ventricular tachycardia, LVEF<45% at first examination, male sex and non-missense mutations (ins-del or splicing) [40]. Contrary to EDMD1, pacemaker is not sufficient to prevent sudden cardiac death, therefore implantation of ICD is recommended. There were several attempts to select serum biomarkers which could help to determine the risk of heart failure progression, to monitor therapy and to determine prognosis. Analysis of selected natriuretic peptides, collagen regulators (MMPs and TIMPs) and extracellular matrix proteins (tenascin C, osteopontin) showed that they indeed fluctuated in both types of EDMD [41], that reflects left ventricle dysfunction and cardiac fibrosis and remodeling, seen in cardiolaminopathy [42], but also in cardiac pathology of other origin. Furthermore, collagen regulators might be influenced not only by myocardial fibrosis, but also by inflammatory and fibrotic changes in skeletal muscles, therefore specificity of those biomarkers in EDMD seems to be low. Irrespectively of the type of EDMD, patients are at greater risk of thromboembolic complications [29,43], mainly cerebral strokes, which tend to occur at younger age than in general population and often aggravate disability caused by muscular dystrophy. In some patients cerebral stroke was a revelator of EDMD [44]. In the past EDMD patients usually died due to a complete cardiac block or malignant arrhythmia. Introduction of heart electrotherapy allowed to control, to some extent, these complications and brought a life-saving option to the treatment of laminopathies with cardiac involvement, however decompensated cardiac failure and cerebral strokes still account for many fatal outcomes. When comparing two groups of patients with Emery-Dreifuss muscular dystrophy, it is clearly visible that clinical course of laminopathy was more severe and less predictable either as muscle or cardiac disease. Consequently, certificate of total inability to work and disability pension are granted more often to patients with EDMD2 than EDMD1, which is associated with worse physical performance as well as more severe cardiac disease – EDMD2 patients with even minimal muscle disease could have cardiac failure which adversely influences not only their ability to undertake and keep the job, but also everyday life [45]. EDMD3 with recessive trait of inheritance, reported at first by Rafaelle di Barletta et al [31], was characterized by early onset severe form of atypical EDMD, with pronounced muscle wasting and contractures, leading to immobilization, but with no cardiac symptoms. Two of 4 patients with EDMD3 from the family described by Jimenez-Escrig et al [46]. also became wheelchair-bound in early adulthood, but supraventricular and ventricular arrhythmia has been found in all four affected individuals.

### Other types of Emery-Dreifuss muscular dystrophy

Laminopathy or emerinopathy is diagnosed in approximately 40% of patients with EDMD-like phenotype. Other known genes associated with the EDMD-like clinical presentation include *SYNE1* and *SYNE2*, encoding nesprin-1 and -2, which are responsible for EDMD4 (OMIM#612998) and EDMD5 (OMIM#612999) [47], respectively. Few cases of EDMD4, reported in the literature, manifested by slowly progressive muscular atrophy and weakness with joint contractures and without significant cardiac abnormalities [48,49]. EDMD5 was characterized by muscle weakness without apparent contractures, arrhythmia and dilated cardiomyopathy with heart failure.

X-linked EDMD6 (OMIM#300696) is associated with mutations in the *FHL-1* gene and manifests clinically as muscle weakness and/or wasting, mainly in scapulo-humeral and pelvic/peroneal regions, joint contractures and rigid spine. However, in a few patients skeletal muscle hypertrophy were seen. Skeletal muscle symptoms usually precede heart involvement, which may manifest as supraventricular and ventricular arrhythmias, conduction defects and cardiac hypertrophy (as opposed to the dilated cardiomyopathy observed in the case of laminopathy), which may be life-threating. In addition, vocal cords palsy with dysphonia, facial involvement, ptosis, and swallowing difficulties may occur. Respiratory failure have been also reported. Female carriers may have heart symptoms, either isolated and/or with mild skeletal muscle involvement. The disease-causing mutations in EDMD6 are distributed in the distal regions of *FHL1* gene and include missense mutations and out-of-frame deletions, which severely affect expression of FHL protein [50].

In 2011 Liang et al [6]. reported two unrelated patients with adult onset atrophy and weakness of proximal muscles accompanied by cardiac conduction defects, associated with heterozygous mutations in *TMEM43* gene, further referred to as EDMD7 (OMIM#614302). Scarcity of the reported cases of EDMD3-7 does not allow to define unified clinical picture of each of those entities. Although they were assigned to the EDMD group, their symptomatology places them anywhere on the spectrum of skeletal muscle laminopathies and rather as EDMD-like than pure EDMD phenotype.

### Non-EDMD skeletal muscle laminopathies

In addition to EDMD, two other main groups of lamin-related skeletal muscle phenotypes have been distinguished: LMNA-related congenital muscular dystrophy (L-CMD; OMIM#613205) [51] which affects young children, and limb-girdle muscular dystrophy type 1 (LGMD1B; OMIM#159001) [52] with adult onset. In the first reports, L-CMD has been divided it into severe form characterized by significant skeletal muscle weakness and extremely delayed motor development, with the onset already in fetal period, and milder form with first symptoms seen before 1^st^ year of life, where affected children were able to sit, but never start walking. In the latter form, axial muscles weakness with head dropping is present, and spine rigidity, hip, knee and Achilles tendon contractures develop relatively early. Elbow contractures are not very pronounced, and in contrast, in some patients elbow hypermobility is seen. Respiratory failure is frequent as a result of respiratory muscle involvement. Cardiac involvement can manifest initially as atrial arrhythmia, preceding development of fast-progressing heart failure [51,53]. Along with a wider availability of new diagnostic techniques, in particular next-generation sequencing, it became clear that L-CMD might be more frequent than it was assumed previously. Initial symptoms might be atypical, starting from floppy infant syndrome or myopathy, sometimes with marked inflammatory component [54]. Further course of the disease may include development of pronounced lumbar hyperlordosis, fast-progressing contractures, feet and hand deformations, which finally lead initially mobile children to wheel-chair bounding [51,55,56]. LGMD1B is characterized by symmetric, proximal weakness of shoulder and hip girdles, which gradually develops in young adults. Contractures are late and not very pronounced. Cardiac involvement includes arrhythmogenic cardiomyopathy with conduction abnormalities, and often precedes skeletal muscle symptoms. It is of note that cardiolaminopathy may also exist as separate entity without skeletal muscle symptoms (cardiomyopathy dilated with conduction defect 1, CMD1A; OMIM#115200) [57]. In patients with dilated cardiomyopathy, laminopathy has been found in 5% and it should be always considered in patients with conduction abnormalities and in families with positive history towards sudden cardiac deaths, especially of young people. Genotype-phenotype correlations showed that patients with cardiolaminopathy without skeletal muscle symptoms or with relatively mild, late-onset muscle involvement more often have been identified to have *LMNA* mutations other than missense [32,36,58]. Skeletal muscle symptoms may be also a component of overlapping syndromes [59], accompanying lipodystrophy and/or metabolic disorders, neuropathy, skin lesions and progeroid features in various combinations [60–64].

Management of patients with EDMD and other laminopathies should include regular cardiological follow-up, considering high penetrance of *LMNA* mutations. Genetic counselling is to be offered to patients and their families, as detection of disease-causing mutations allows to initiate preventive measures also in female carriers of *EMD* or *FHL-1* and in asymptomatic carriers of *LMNA* mutations, which might be at high risk of life-threatening cardiological complications [65–67].

## Abbreviations

*AVB*: atrio-ventricular block
*CK*: creatine kinase
*CMD1A*: cardiomyopathy dilated with conduction defect 1
*EDMD*: Emery-Dreifuss muscular dystrophy
*FHL-1*: four and a half LIM domains 1
*ICD*: implantable cardioverter-defibrillator
*LAP*: lamina-associated polypeptide
*L-CMD*: LMNA-related congenital muscular dystrophy
*LGMD1B*: limb-girdle muscular dystrophy type 1B
*MMPs*: matrix metalloproteinases
*SCD*: sudden cardiac death
*TIMPs*: tissue inhibitors of matrix metalloproteinases
*TMEM43*: transmembrane protein 43
